# Promoter engineering enables overproduction of foreign proteins from a single copy expression cassette in *Bacillus subtilis*

**DOI:** 10.1186/s12934-019-1159-0

**Published:** 2019-06-14

**Authors:** Chaoyang Zhou, Bin Ye, Shan Cheng, Leizhen Zhao, Yuanxin Liu, Jiandong Jiang, Xin Yan

**Affiliations:** Department of Microbiology, College of Life Sciences, Key Laboratory for Microbiological Engineering of Agricultural, Environment of Ministry of Agriculture, Nanjing Agricultural University, 6 Tongwei Road, Nanjing, 210095 Jiangsu People’s Republic of China

**Keywords:** *Bacillus subtilis*, Promoter engineering, Chromosomal integration, Highly expression

## Abstract

**Background:**

*Bacillus subtilis* is developed to be an attractive expression host to produce both secreted and cytoplasmic proteins owing to its prominent biological characteristics. Chromosomal integration is a stable expression strategy while the expression level is not ideal compared with plasmid expression. Thus, to meet the requirement of protein overexpression, promoter, as one of the key elements, is important. It is necessary to obtain an ideal promoter for overproduction of foreign proteins from a single copy expression cassette.

**Results:**

The activity of promoter *P*_*ylb*_ was further enhanced by optimizing the − 35, − 10 core region and upstream sequence (UP) by substituting both sequences with consensus sequences. The final engineered promoter exhibited almost 26-fold in β-galactosidase (BgaB) activity and 195-fold in super-folded green fluorescent protein (sfGFP) intensity than that of WT. The two proteins account for 43% and 30% of intracellular proteins, respectively. The promoter was eventually tested by successful extracellular overproduction of Methyl Parathion Hydrolase (MPH) and Chlorothalonil hydrolytic dehalogenase (Chd) to a level of 0.3 g/L (144 U/mL) and 0.27 g/L (4.4 U/mL) on shake-flask culture condition.

**Conclusions:**

A strong promoter was engineered for efficient chromosomally integrated expression of heterologous proteins.

**Electronic supplementary material:**

The online version of this article (10.1186/s12934-019-1159-0) contains supplementary material, which is available to authorized users.

## Background

*Bacillus subtilis*, a species of Gram-positive aerobic soil bacteria, is an attractive industrial workhorse for production of various enzymes and industrial recombinant proteins due to its GRAS (generally recognized as safe) status, well-characterized protein secretion mechanisms and large-scale fermentation processes [1–5]. In addition, the bacterium has no significant bias in codon usage and efficient genetic manipulation is available [6, 7]. Thus, more attention has been paid to its expression systems for the purpose of the commercial application and basic research.

Plasmid-mediated recombinant production of proteins in bacteria is unstable during the late stage of fermentation [8]. Moreover, the safety concerns and legal requirements surrounding the use of antibiotic is another bottleneck in food industry. Chromosomal integration offers a more stable alternative to maintenance of foreign inserted expression cassette. However, the expression level may not meet the requirement when compared with that of multi-copy plasmid expression. Protein production was mainly determined by transcription, translation and post-translation level. To realize efficient chromosomally integrated protein expression, promoter is of great importance in transcription level because it directly affects the efficient synthesis of fundamental transcripts. Therefore, a powerful promoter is desirable to drive gene overexpression.

Nowadays, existing promoters can be generally divided into three major groups: constitutive promoters [9–13], inducible promoters [14–25] and stationary phase promoters [26–28]. Recently, a stationary phase promoter *P*_*ylb*_ [29] was identified. Based on the *P*_*ylb*_ promoter, in this work, a new strong promoter was developed to realize high level protein expression through single-copy expression cassette integration.

## Results

### Assessment of the WT promoter *P*_*ylb*_

**Fig. 1:**
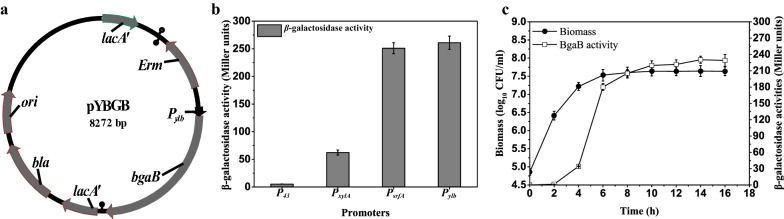
**a** Construction of the integrative plasmid pYBGB. **b** Comparison of the maximum yield of BgaB under the control of different promoters. All cultures were grown in triplicate, and each experiment was performed at least twice. Error bars indicate standard deviations. **c** The expression level and pattern of BgaB measured in strain WBBgaB. During 24 h of cultivation, cells were sampled periodically and analyzed by examining the biomass and BgaB activity

To begin with, WT promoter was compared with commonly used constitutive promoter *P*_*43*_ [9], inducible promoter *P*_*xylA*_ [14], and stationary phase promoter *P*_*srfA*_ [27]. The expression level of reporter protein BgaB was used to reflect the strength of the promoters. Promoter *P*_*ylb*_ was prominent for its strength and stationary phase (Fig. 1b, c and Additional file 1: Figures S1, S2). During the lag phase and the early exponential phase, there was little BgaB activity detected. The reporter protein began to emerge at the mid-exponential phase; the activity sharply increased to the peak value during the transition to stationary phase, and remained constant during the followed stationary phase (Fig. 1c). Thus, the WT *P*_*ylb*_ is deserved to be further engineered for overexpression of proteins in *B. subtilis*.

### Engineering the core regions to improve transcription

**Fig. 2:**
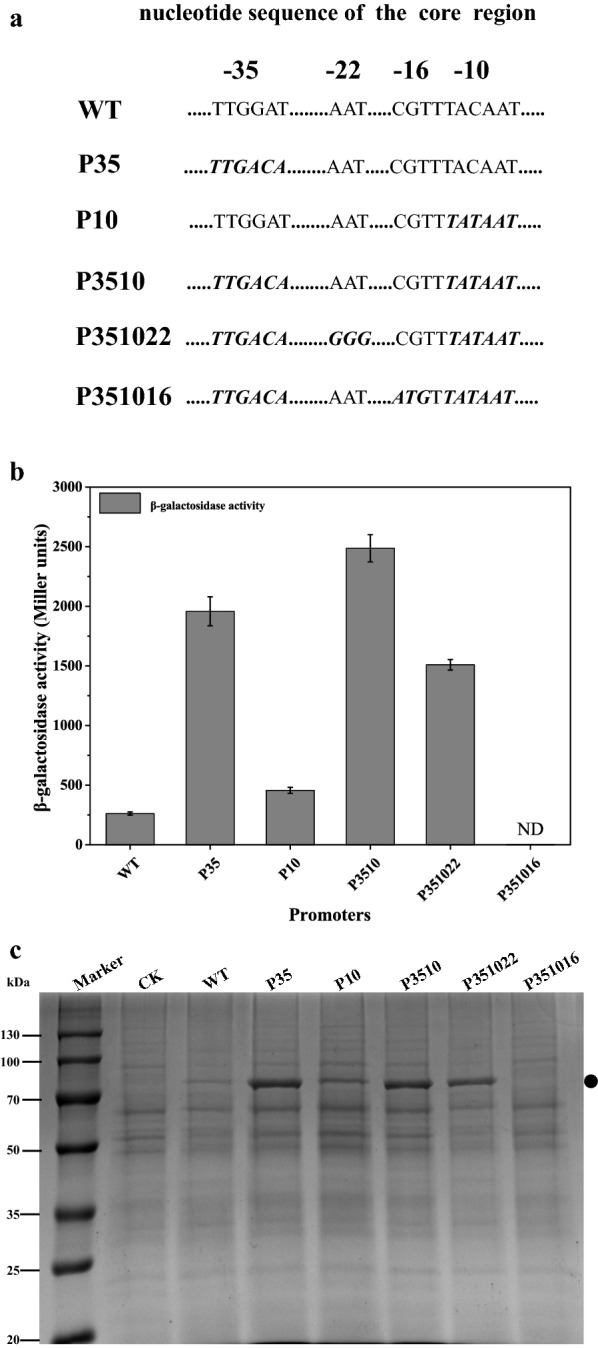
Optimization of the core region of the *P*_*ylb*_ promoter. **a** the core regions of the *P*_*ylb*_ promoter were changed to corresponding consensus sequence. The nucleotides in bold italic indicate mutated sequences. **b** The BgaB expression level under the control of *P*_*ylb*_ derivatives. **c** SDS-PAGE analysis of the BgaB expression. Equal amounts (30 μg) of total protein were loaded into each lane. The band corresponding to BgaB was marked. All cultures were grown in triplicate, and each experiment was performed at least twice. Error bars indicate standard deviations. CK represents the intracellular protein of strain WB800

− 35 and − 10 regions were the most important sequences in promoter strength and the regions were widely engineered to enhance promoter transcription [30]. Thus, the − 10 and − 35 regions of *P*_*ylb*_ were changed into the corresponding consensus sequence separately or in combination (Fig. 2a). The reporter protein BgaB was used to assess the strength of the engineered promoters. A promoter with the consensus − 10 hexamer (P10) was 0.5-fold stronger than *P*_*ylb*_ while the BgaB expression level controlled by the *P35* promoter resulted in a sevenfold increase (Fig. 2b, c). When both changes were combined into one single promoter (*P3510*), its activity was enhanced about ninefold (Fig. 2b, c and Additional file 1: Figures S3, S4). In addition to − 35 and − 10 regions, − 16 and − 22 region will also influence promoter strength [30, 31]. Next, − 16 region was also changed into the corresponding consensus sequence TRTG (where R stands for A or G) based on *P3510*, generating *P351016* (Fig. 2a). However, BgaB activity could not be detected under the control of *P351016* (Fig. 2b, c and Additional file 1: Figures S3, S4). The activity of promoter with mutation in the − 22 region (Fig. 2a) decreased slightly under the control of *P351022* (Fig. 2b, c and Additional file 1: Figures S3, S4).

### Engineering the upstream sequence to improve transcription

**Fig. 3:**
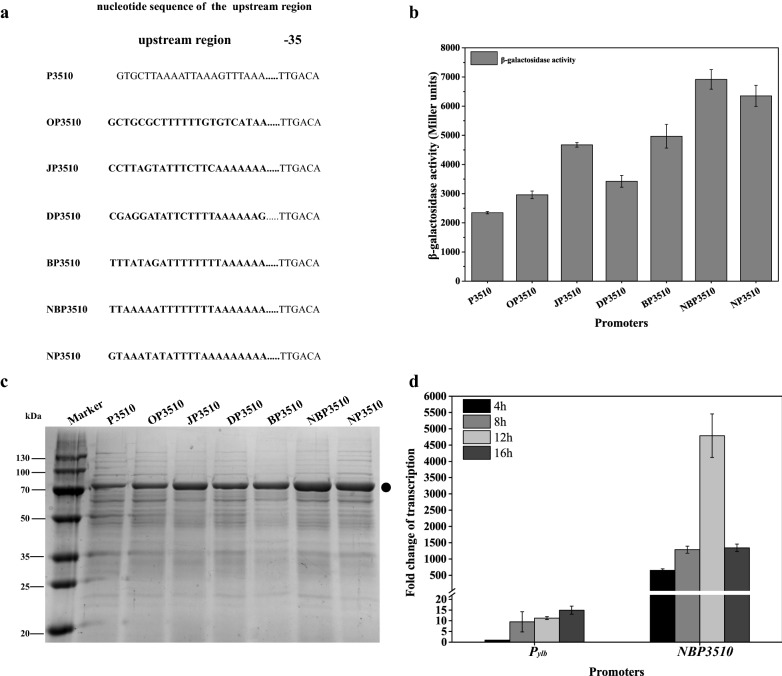
Optimization of the upstream region of the *P3510* promoter. **a** the upstream regions of the *P3510* promoter were changed to corresponding sequence. The nucleotides in bold italic indicate mutated sequences. **b** The BgaB expression level under the control of *P3510* derivatives. **c** SDS-PAGE analysis of the BgaB expression. Equal amounts (30 μg) of total protein were loaded into each lane. The band corresponding to BgaB was marked. **d** Comparative analysis of the transcription levels of two promoters at different time points. The transcription level of *P*_*ylb*_ promoter at the time point 4 h was set as 1. The reference gene was 16 s rRNA. All cultures were grown in triplicate, and each experiment was performed at least twice. Error bars indicate standard deviations

Since upstream elements could enhance transcription initiation in *B. subtilis* [31, 32], to further enhance the promoter *P3510*, putative UP elements (− 59 to − 38) of four *rrn* operons (*rrnO*, *rrnJ*, *rrnD*, *rrnB*) controlled by tandem promoters (P1 and P2) [33] were introduced to replace the native region (Fig. 3a). As shown in Fig. 3b, four UP elements showed a distinct activation of the transcription as compared to the native UP element. The engineered *BP3510* and *JP3510* was approximately one fold stronger than that of the *P3510* promoter, demonstrating the strong stimulation of the promoter activity. When the UP element of *rrnB* was engineered to a consensus sequence [34], − 59 nnAAA(A/T)(A/T)T(A/T)TTTTnnAAAAnnn − 38, the new UP element was mutated to TTAAAAATTTTTTTTAAAAAAA (Fig. 3a). The mutant promoter *NBP3510* showed superior activity to all of the other promoters and the BgaB expression was twofold higher than that of the *P3510* (Fig. 3b, c and Additional file 1: Figures S5, S6) and enhanced by 26-fold compared with that of the WT promoter. In addition, the native UP region of *P3510* was also changed to a consensus sequence (Fig. 3a) and the resulting promoter *NP3510* was enhanced up to 1.7-fold (Fig. 3b, c and Additional file 1: Figures S5, S6). In summary, the strongest promoter *NBP3510* allowed intracellular accumulation up to about 43% of the total cellular protein. In addition, promoter engineering had no significant effect on bacterial growth (Additional file 1: Figures S14, S15 and Table S2). Thus, the increased production was mainly due to the improved transcription. Western blot results showed that BgaB was expressed (Additional file 1: Figures S11 and S13). Furthermore, qRT-PCR was used to verify the transcription level of *NBP3510*. RNA was extracted after 4, 8, 12, 16 h. The highest transcription level of *NBP3510* was 340-fold stronger than promoter *P*_*ylb*_ (Fig. 3d).

Although the strength of *NBP3510* was dramatically enhanced at both log phage and stationary phase after engineering, it still exhibits the property of “stationary phase” (Fig. 1c and Additional file 1: Figures S5, S6). Since target gene was also transcribed in the early stage of cell growth under the control of stationary-phase promoter. Thus, the kinetics of BgaB production were not fit very well with Luedeking and Piret [35] (Additional file 1: Figure S17 and Table S3).

### Intracellular expression of the sfGFP protein

**Fig. 4:**
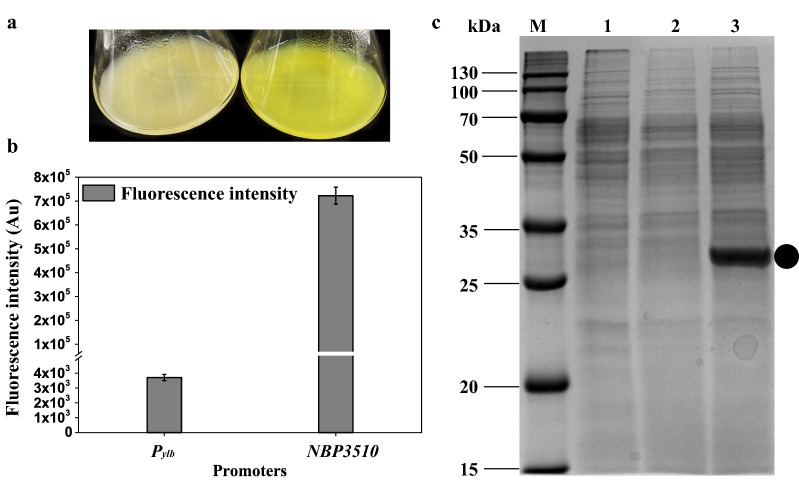
Intracellular expression of sfGFP. **a** The fluorescence imagines of strains WBGFP (*P*_*ylb*_) and WBSGFP (*NBP3510*). Strains were cultured for 16 h in LB medium and imagines were taken. **b** The fluorescence intensity controlled by different promoters. **c** The accumulative sfGFP protein in different strains. M, Marker. Lane 1, Strain WB800. Lane 2, Strain WBGFP. Lane 3, Strain WBSGFP. Equal amounts (30 μg) of total protein were loaded into each lane. The band corresponding to sfGFP was marked. All cultures were grown in triplicate, and each experiment was performed at least twice. Error bars indicate standard deviations

Another reporter protein sfGFP [36] was used to verify if the strong promoter *NBP3510* was suitable for highly efficient intracellular expression. The *NBP3510* showed prominent fluorescence by naked-eye detection (Fig. 4a) and the fluorescence intensity was enhanced up to 195-fold than that of the WT promoter (Fig. 4b and Additional file 1: Figures S7, S8). The sfGFP expression reached 30% of total cellular protein in SDS-PAGE (Fig. 4c). Western blot results showed that sfGFP was expressed (Additional file 1: Figures S11 and S13). Together with the BgaB expression, these results revealed that the engineered promoter *NBP3510* was sufficient for efficient chromosomally integrated intracellular expression.

### Extracellular expression of Methyl Parathion Hydrolase (MPH) and Chlorothalonil hydrolytic dehalogenase (Chd) by promoter *NBP3510*

**Fig. 5:**
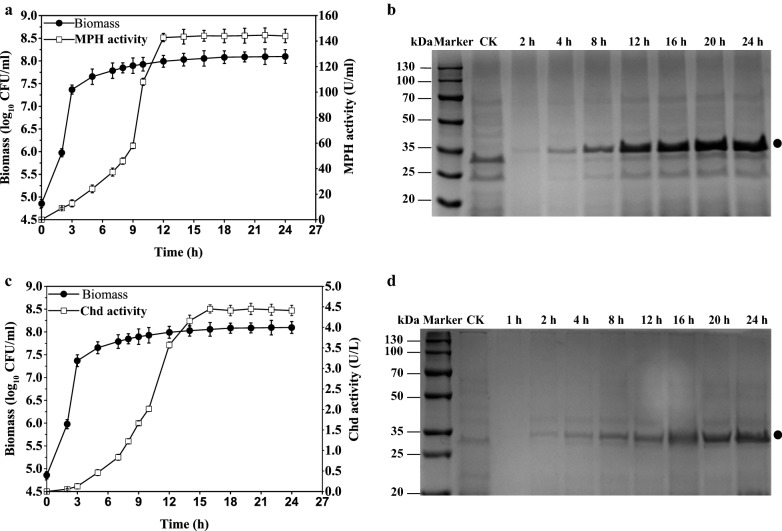
Overproduction of Methyl Parathion Hydrolase (MPH) and Chlorothalonil hydrolytic dehalogenase (Chd) using the promoter *NBP3510*. **a** The activities of MPH in the supernatant (solid rectangle) and cell density (empty circle) were determined at different times. **b** The expression of MPH was analyzed by SDS-PAGE. Equal amounts (20 μL) of culture supernatant were loaded into each lane. **c** The activities of Chd in the supernatant (solid rectangle) and cell density (empty circle) were determined at different times. **d** The expression of Chd was analyzed by SDS-PAGE. Equal amounts (10 μL) of culture supernatant were loaded into each lane. The bands indicating to the target proteins were marked. All cultures were grown in triplicate, and each experiment was performed at least twice. Error bars indicate standard deviations. CK represents the extracellular protein of strain WB800

To test whether the engineered promoter was suitable for overproduction of extracellular protein, strains WBSMPH and WBSChd were cultured in 2 × SR [37] medium. The cell growth, MPH activity, Chd activity and protein overproduction were measured throughout cultivation (Fig. 5 and Additional file 1: Figures S9, S10). Mutant strains showed no significant difference on bacterial growth compared with strain WB800 (Additional file 1: Figure S16 and Table S2). The extracellular protein displayed stationary phase-dependent pattern and the activity was significantly increased from the mid-exponential phase to stationary phase (Fig. 5a, c). The activity of MPH measured from the supernatant was as high as 144 U/ml (Fig. 5a, b) which was 5.3-fold of that of plasmid pP43NMK-mediated expression (*P*_*43*_-*mpd* cassette) [38]. The yield of MPH was increased to 0.3 g/L on shake-flask culture condition. The activity of Chd measured from the supernatant was as high as 4.4 U/mL (Fig. 5c, d) which was 300-fold higher than that of plasmid pP43Chd-mediated expression (*P*_*43*_-*chd* cassette) [39]. The yield of Chd was increased to 0.27 g/L on shake-flask culture condition. Western blot results showed that MPH and Chd were expressed (Additional file 1: Figures S12, S13).

## Discussion

So far, most promoters reported in *B. subtilis* were ligated to plasmid for protein production [19, 22, 27, 40–43]. Only four promoters (*cry3Aa* [26], *amyQ* [44], *aprE* [45] and a hybrid promoter [46] consist of *P*_*43*_ [9], *P*_*ylb*_ [29] and *P*_*rha*_ [47] were integrated to express single-copy gene. However, detailed protein production level was not mentioned except promoter *aprE* (10% intracellular total protein). Since single copy of expression cassette was not comparable to plasmid-expression system, it is necessary to choose an ideal promoter for stable and efficient protein production.

To date, promoters could be obtained by three fundamental approaches: (1) screening from the microbial genome and identification of the promoter [48–52]; (2) generating libraries of artificial promoters [36, 53–56]; (3) engineering the core region of known promoters [26, 28, 31, 45]. In this work, existing stationary phase-dependent promoter *P*_*ylb*_ was engineered to achieve protein overproduction in a single copy cassette.

It is widely recognized that core region and the UP element were the key elements that contribute most to promoter activity. By engineering the core region to consensus sequence, the results indicated − 35 region played a pivotal role in promoter activity, which was in accordance with previous findings that the promoter activity of *P*_*aprN*_ and *P*_*groES*-*groEL*_ were improved by modifying the − 35 region [31, 57]. It is presumably the poorer homology of the − 35 motifs than the − 10 region in *P*_*ylb*_ to δ^A^-dependent consensuses that resulted in the major contribution of the − 35 region. The − 16 region was reported to have a pronounced effect on transcription [31] when changed to consensus sequence TRTG and the TRTG motif was shown to stabilizes the transcription initiation open complex [58]. However, our result was opposed to the report and no BgaB activity was detected while engineering the − 16 region. It is probably that the native − 16 region is a key sequence involved in transcriptional activity. This result may be the most difference compared with other promoters. The − 22 region was also a potential target [31]. The phenomenon that the activity of promoter decreased slightly when changed from AAT to GGG indicated that “AT base” maybe suitable for “G” at positions − 22 region.

The UP element, a component of bacterial promoters located upstream of the − 35 hexamer, increases transcription by interacting with the RNA polymerase *α*-subunit [34]. It has been reported that UP element could stimulate transcription initiation in *B. subtilis* although UP elements are not crucial for transcription of all promoters [32]. Since the major products of all cellular transcription in *B. subtilis* are rRNA and tRNA, which constitute more than 95% of the total RNA [59], the UP elements from *rrn* operator may further stimulate promoter activity. There are 10 *rrn* operons in *B. subtilis*, controlled by tandem (*P1* and *P2*) promoters. According to previously reported *rrn* operons [33], we chose UPs from four strong *rrn* operons controlled by P1 promoter. While replacing the UP sequence with that of *rrnB* operon, the promoter activity showed the best performance compared to other three. This is consistent with the report that strong *rrnB P1* UP element increased the rate of RNAP binding [60]. To further engineering the promoter, the UP of *rrnB* was changed to consensus sequence, generating a new UP element consisting of A and T only. The UP engineering was verified again that the activity of a natural UP element could be improved by making it more AT-rich [34].

In this study, two reporter proteins BgaB and sfGFP were highly expressed. The intracellular expression level (43%) of BgaB was higher than that driven by promoter *P*_*grac*_ in plasmid pHT100 (30%) [31] and the intracellular expression level (30%) of sfGFP was comparable to that driven by promoter *P*_*srfA*_ in plasmid pBSG3x (28.4%) [28]. The expression of two intracellular single-copy reporter proteins demonstrated that promoter *NBP3510* was suitable for intracellular protein expression. To further exploit the application of *NBP3510* promoter in *B. subtilis*, MPH and Chd proteins were extracellular expressed. The activity of MPH (144 U/mL) was higher than that driven by promoter *P*_*43*_ in plasmid pP43NMK (27.1 U/mL) and the expression level (0.3 g/L) was also superior to pP43NMK-mediated expression (53 mg/L) [9]. The activity of Chd (4.4 U/mL) was higher than that driven by promoter *P*_*43*_ in plasmid pP43Chd (14.5 U/L) and the expression level (0.27 g/L) was also superior to pP43Chd-mediated expression (5.65 mg/L) [39]. All in all, all these results indicate that single copy of promoter *NBP3510* could be used to overexpress foreign proteins in *B. subtilis*.

Aside from promoter strength, there are still many variables affecting expression levels, including the stability of the mRNA [61], protein translation [62, 63], the culture conditions [64, 65] and so on. Thus, protein production can be further improved by optimizing the variables above.

## Conclusions

*Bacillus subtilis*, a versatile microorganism, has been used to overexpress various recombinant proteins owing to its outstanding biological characteristics. In this study, highly efficient promoter *NBP3510* was generated and two intracellular proteins (BgaB, sfGFP) and extracellular proteins (MPH, Chd) were overexpressed from a single copy expression cassette.

## Methods

### Strains, plasmids and growth conditions

Bacterial strains and plasmids used in this study are listed in Table 1. Plasmid pAX01 was a gift from the Bacillus Genetic Stock Center (BGSC). *Escherichia coli* Top 10 were used as the host for gene cloning. *B. subtilis* WB800 was used for gene expression and integration. Unless otherwise indicated, the final concentrations of antibiotics were as follows, mg/L: ampicillin (Amp), 100 for *E. coli*; erythromycin (Em), 5 for *Bacillus*. Strains were cultivated in Luria–Bertani (LB) medium or 2 × Super-Rich (SR) [37] medium. LB medium consisted of 1% tryptone, 0.5% yeast extract and 0.5% NaCl while 2 × SR medium consisted of 3% tryptone, 5% yeast extract and 0.6% K_2_HPO_4_, pH 7.2. Strains were cultivated at 37 °C in shaking flasks (SHUNIU, GG-17, Sichuan SHUBO Co., LTD, China) on an incubator shaker (IS-RDV1, Crystal, China) operating at 200 rpm. To express recombinant protein, 0.3 mL of preculture was transferred into 250-mL shaking flasks that were loaded with 30 mL of LB or 2 × SR liquid medium. The percentages of the produced intracellular proteins were calculated by Imagine J software. The cell density was determined by measuring the OD_600_ with a UV-1800/PC spectrophotometer (Shanghai, MAPADA Instrument Co., Ltd., China).

**Table 1:** Strains and plasmids used in this study

Strain or plasmid	Characteristics^a^	Source or reference^b^
Plasmids		
pAX01	*P*_*xylA*_, Ap^r^, Em^r^, *lacA*	[15]
pYBGB	pAX01 containing *P*_*ylb*_-*bgaB* cassette	This work
pLJ-2	Cm^R^; *E. coli*-*Bacillus* shuttle vector	[19]
pUS20	Spc^r^, unstable in *B. subtilis, E. coli*-*Bacillus* shuttle vector	[71]
Strains		
*E. coli* Top10	F^−^ *mcrA* Δ(*mrr*-*hsdRMS*-*mcrBC*) ϕ80*lacZ*ΔM15 Δ*lacX74 recA1 araD139* Δ(*ara leu*)7697 *galU galK endA1 nupG*	TransGene Biotech (Beijing, China)
*B. subtilis*		
168	*trpC2*	BGSC 1A1
WB800	(168) Δ*nprE*, Δ *aprA*, Δ*epr*, Δ *bpr*, Δ*mpr*, Δ*nprB*, Δ*vpr*, Δ*wprA*	[72]
WBEmBgaB	Strain WB800 derivate, *lacA*::*P*_*ylb*_-*bgaB*, Em^r^	This work
WBBgaB	Strain WB800 derivate, *lacA*::*P*_*ylb*_-*bgaB*	This work
BS43	Strain WB800 derivate, *lacA*::*P*_*43*_-*bgaB*	This work
BSxylA	Strain WB800 derivate, *lacA*::*P*_*xylA*_-*bgaB*	This work
BSsrfA	Strain WB800 derivate, *lacA*::*P*_*srfA*_-*bgaB*	This work
35BgaB	Strain WB800 derivate, *lacA*::*P35*-*bgaB*	This work
10BgaB	Strain WB800 derivate, *lacA*::*P10*-*bgaB*	This work
3510BgaB	Strain WB800 derivate, *lacA*::*P3510*-*bgaB*	This work
351016BgaB	Strain WB800 derivate, *lacA*::*P351016*-*bgaB*	This work
351022BgaB	Strain WB800 derivate, *lacA*::*P351022*-*bgaB*	This work
OBgaB	Strain WB800 derivate, *lacA*::*OP3510*-*bgaB*	This work
JBgaB	Strain WB800 derivate, *lacA*::*JP3510*-*bgaB*	This work
DBgaB	Strain WB800 derivate, *lacA*::*DP3510*-*bgaB*	This work
BBgaB	Strain WB800 derivate, *lacA*::*BP3510*-*bgaB*	This work
WBSBgaB	Strain WB800 derivate, *lacA*::*NBP3510*-*bgaB*	This work
WBGFP	Strain WB800 derivate, *lacA*:: *P*_*ylb*_ -*sfGFP*	This work
WBSGFP	Strain WB800 derivate, *lacA*::*NBP3510*-*bgaB*	This work
WBSMPH	Strain WB800 derivate, *lacA*::*NBP3510*-*mpd*	This work
WBSChd	Strain WB800 derivate, *lacA*::*NBP3510*-*chd*	This work

^a^Ap^R^: ampicillin resistance; Cm^R^: chloramphenicol resistance; Em^R^: erythromycin resistance; Spc^r^: spectinomycin resistance

^b^BGSC: *Bacillus* Genetic Stock Center

### DNA manipulation techniques

Oligonucleotides synthesis (Additional file 1: Table S1) and DNA sequencing were performed by Sangon Biotech Co., Ltd. (Shanghai, China). The isolation and manipulation of recombinant DNA was performed using standard techniques. All enzymes were commercial preparations. Phusion DNA high-fidelity polymerase was purchased from NEB (Shanghai, China). The transformation of *B. subtilis* was carried out as previously described [66].

### Comparison of the WT promoter *P*_*ylb*_ with other promoters in BagB expression

To construct *P*_*ylb*_-driven bagB expression strain, Fragment 1, a 2.0-kb fragment, comprising the *bgaB* encoded β-galactosidase activity was cloned from plasmid pLJ-2 [19] using the primer pair P1/P2. Concomitantly, Fragment 2, carrying the *P*_*ylb*_ promoter from *B. subtilis* 168 genome was generated using the primer pair P3/P4. The third fragment, the pAX01 plasmid backbone was amplified using the primer pair P5/P6. The terminuses of three fragments were flanked by 30-bp homology in order using the Sequence and Ligation Independent Cloning (SLIC) method, yielding integrating plasmid pYBGB (Fig. 1a). The plasmid was then transformed into *B. subtilis* WB800, resulting in strain WBEmBgaB.

To eliminate the Em resistance, left flanking region (LF) and the right flanking region of *erm* was fused together using the primer pairs P7/P8 and P9/P10. The fragment was transformed to *B. subtilis* WBEmBgaB with the temperature-sensitive plasmid pUS20 by nature co-transformation. The temperature-sensitive plasmid pUS20 was subsequently cured by overnight growth without selection, generating the marker-free strain WBBgaB.

As for other promoter-driven expression strains, co-transformation was applied to replace the promoters with the *P*_*ylb*_ promoters in strain WBBgaB. First, the promoters *P*_*43*_, *P*_*xylA*_ and *P*_*srfA*_ were amplified from *B. subtilis* 168 genome using primer pairs P11/P12, P13/P14 and P15/P16. Next, the corresponding LF region and RF region were amplified from strain WBBgaB using the primer pairs P7/P17, P7/P19, P7/P21 and P18/P10, P20/P10, P22/P10, respectively. The promoters were flanked by the corresponding LF and RF region by overlapping-extension PCR. Three fused fragments with promoter-driven bgaB expression cassettes were separately transformed to *B. subtilis* WB800 by nature co-transformation described above. The resulting strains were designed as BS43, BSxylA and BSsrfA. All of the target mutation was confirmed by PCR amplification followed by DNA sequencing.

### Site-mutation of the WT *P*_*ylb*_ promoter

To mutate the core region of the *P*_*ylb*_ promoter, for instance, the − 35 region, the strain WBBgaB containing *P*_*ylb*_-bgaB cassette was used as template. Primer pairs P7/P23 and P24/P10 were used to amplify the LF region and RF region, respectively. The mutation was introduced into the primers P23 and P24. Primers P23 and P24 were reverse complementation. The LF and RF fragments were fused by overlapping PCR and co-transformed with the plasmid pUS20 followed by elimination of pUS20, generating the strain 35bgaB. Other mutant promoters, P10, P3510, P351016 and P351022 were also constructed using the method described above and the corresponding strains (10BgaB, 3510BgaB, 351016BgaB and 351022BgaB) are also constructed using the primers listed in Additional file 1: Table S1.

As for mutation of the upstream sequence of *P3510*, strain 3510bgaB was used as template. The native UP was replaced by four upstream sequences of *rrn* operon (*rrnO*, *rrnJ*, *rrnD*, *rrnB*) P1 promoter [33]. New promoters *OP3510*, *JP3510*, *DP3510*, *BP3510* and *NBP3510* were generated the same as above. The corresponding strains (OBgaB, JBgaB, DBgaB, BBgaB and WBSBgaB) and the primers are listed in Additional file 1: Table S1.

### Measurement of BgaB encoded β-galactosidase activity

*Bacillus subtilis* WB800 containing each engineered promoter with the *bgaB* reporter gene was cultured at 37 °C in an orbital shaker at 200 rpm in LB medium without antibiotic. After incubation for 16 h, samples were taken for determination of β-galactosidase activities. The β-galactosidase specific activities were converted to Miller units, as described previously [67]. The values shown are the average of three independent experiments.

### Sodium dodecyl sulfate polyacrylamide gel electrophoresis (SDS-PAGE)

The protein samples were mixed with 5 × SDS-PAGE sample buffer (125 mM Tris–HCl pH 6.8, 4% SDS, 20% glycerol, 10% β-mercaptoethanol and 0.004% bromophenol) and heated at 100 °C for 10 min. The sample was centrifuged at 12,000 rpm for 5 min and the supernatant was used for SDS-PAGE. The electrophoresis was performed at 80 V through the stacking gel (5%) and at 120 V through the separation gel (9%) until the bromophenol blue marker dye reached to within 0.5 cm of the bottom of the gel. Then, the protein bands were stained with Coomassie Brilliant Blue R-250. After staining, gels were destained overnight in a solution containing 5% ethanol and 10% acetic acid.

### Construction of the GFP, Methyl Parathion Hydrolase (MPH) and Chlorothalonil hydrolytic dehalogenase (Chd) expression strains

To construct integrative strain for intracellular GFP expression and extracellular MPH and Chd expression, *sfgfp* [36] was synthesized from Genescript Company (Nanjing, China). Gene *mpd* encoding methyl parathion hydrolase was amplified from the plasmid pP43NMK [38] using primer pairs P43/P44. Gene *chd* encoding chlorothalonil hydrolytic dehalogenase was amplified from the plasmid pP43Chd using primer pairs P45/P46 [39]. For extracellular expression of MPH and Chd, the single peptide of *aprE* was assembled to *mpd* and *chd* using primer pairs P47/P48 and P49/P50. The corresponding LF (including the promoter *P*_*ylb*_ or *NBP3510*) region was cloned from the strain WBBgaB and WBSBgaB. The common RF region was amplified from the strain WB800. Then the LF (*P*_*ylb*_ or *NBP3510*), sfGFP and the RF fragment were fused together and transformed to WB800, generating the strains WBGFP and WBSGFP. Strains harboring *NBP3510*-*mpd* cassette and *NBP3510*-*chd* were also constructed as described above, namely WBSMPH and WBSChd, respectively.

### Flow cytometric analysis

*Bacillus subtilis* WB800 with the *sfGFP* reporter gene was cultured at 37 °C in an orbital shaker at 200 rpm in LB medium without antibiotic. Cells were taken and washed twice with phosphate-buffered saline (PBS, pH 7.5), diluted tenfold in PBS and then analyzed with a BD Accuri C6 flow cytometer (BD, Oxford, UK) using an argon laser at 488 nm as described previously [68]. For each sample, at least 5 × 10^4^ cells were analyzed. Standard deviations are based on a minimum of three statistically independent experiments. Data were obtained using FlowJo V10 software (http://www.flowjochina.com/).

### Expression of MPH and Chd using promoter *NBP3510*

A fresh overnight culture of the recombinant strain containing *mpd* or *chd* cassette was inoculated into 250-mL shake flasks containing 30 mL 2 × SR [37] liquid medium, cultivated 24 h and periodically sampled. A cell-free supernatant was obtained by centrifugation (5 min, 10,000×*g*). MPH activity measurements were performed as previously described [38, 69]. One unit of MPH activity was defined as the amount of enzyme required to hydrolyze 1 μmol methyl parathion in 1 min at 35 °C. Chd activity measurements were performed as previously described [39, 70]. One unit of Chd activity was defined as the amount of enzyme needed to decrease 1 mmol chlorothalonil per minute under optimal conditions.

## Additional file

**Additional file 1. Fig. S1–S2** The expression pattern of BgaB in mutant strains with different promoters. **Fig. S3–S4** The expression pattern of BgaB controlled by mutant *P*_*ylb*_. **Fig. S5-S6** The expression pattern of BgaB controlled by mutant *P3510*. **Fig. S7–S8** The expression pattern of sfGFP in mutant strains. **Fig. S9–S10** The control of MPH (a) and Chd (b) expression in strain WB800. **Fig. S11** Purification of BgaB and sfGFP. **Fig. S12** Purification of MPH and Chd. **Fig. S13** Verification of BgaB, sfGFP, MPH and Chd by western blot. **Fig. S14-S16** The kinetics of bacterial growth. **Fig. S17** The kinetic model for BgaB production in mutant strains. **Table S1** Primers used in this study. **Table S2** the parameters in the kinetics of bacterial growth. **Table S3** the parameters in the kinetics of BgaB production.

## Data Availability

All data generated or analyzed during this study are included in this published article.

## Abbreviations

BGSC: Bacillus Genetic Stock Center
SLIC: sequence and ligation independent cloning
MPH: Methyl Parathion Hydrolase
Chd: chlorothalonil hydrolytic dehalogenase
sfGFP: super-folded green fluorescent protein
UP: upstream sequence
