# Supplementary material for: Promoter engineering enables overproduction of foreign proteins from a single copy expression cassette in Bacillus subtilis
Source: Microb Cell Fact. 2019 Jun 14;18:111. doi: 10.1186/s12934-019-1159-0 (PMC6570832; doi:10.1186/s12934-019-1159-0)
Supplement: Supplementary file 1 — Additional file 1. Fig. S1–S2 The expression pattern of BgaB in mutant strains with different promoters. Fig. S3–S4 The expression pattern of BgaB controlled by mutant Pylb. Fig. S5-S6 The expression pattern of BgaB controlled by mutant P3510. Fig. S7–S8 The expression pattern of sfGFP in mutant strains. Fig. S9–S10 The control of MPH (a) and Chd (b) expression in strain WB800. Fig. S11 Purification of BgaB and sfGFP. Fig. S12 Purification of MPH and Chd. Fig. S13 Verification of BgaB, sfGFP, MPH and Chd by western blot. Fig. S14-S16 The kinetics of bacterial growth. Fig. S17 The kinetic model for BgaB production in mutant strains. Table S1 Primers used in this study. Table S2 the parameters in the kinetics of bacterial growth. Table S3 the parameters in the kinetics of BgaB production. [file 12934_2019_1159_MOESM1_ESM.pdf]

Additional materials

Methods:

Construction of 6×His tag strains

The 6×His tag was fused to C-terminal of four proteins (BgaB, sfGFP, MPH and Chd) using the genome of strain WBSBgaB, WBSGFP, WBSMPH and WBSChd as template, respectively. The primers used were list in supplementary Table S1. The mutant strains were named as WBSBgaBH, WBSGFPH, WBSMPHH and WBSChdH.

Western blot analysis

Briefly, the purified proteins were separated on 9% SDS-PAGE gels, and subsequently transferred to nitrocellulose membranes. The primary antibodies were anti-6×His rabbit antibody (Sangon Biotech, China, NO. D110002). The secondary antibodies were HRP-conjugated Mouse anti-rabbit IgG (Sangon Biotech, China, NO. D110065). The signals were detected using the Pro-Light HRP Chemiluminescent Kit (Tiangen Biotech).

The kinetics of bacterial growth

The kinetics of bacterial growth was described by the logistic equation [1].

$$\frac{dx}{dt} = \mu_m \left(1 - \frac{x}{x_m}\right) \quad (1)$$

$$\mathrm{x} = \frac{x_0 x_m e^{\mu_m t}}{x_m - x_0 + x_0 e^{\mu_m t}} \quad (2)$$

The kinetic model for enzyme production

The kinetic model for enzyme production was described by the Luedeking-Piret equation [2].

$$\frac{dp}{dt} = \alpha \frac{dx}{dt} + \beta x \quad (3)$$

$$P = P_0 + \alpha \frac{x_0 (x_m - x_0)(e^{\mu_m t} - 1)}{x_m - x_0 + x_0 e^{\mu_m t}} + \beta \frac{x_m}{\mu_m} \ln \frac{x_m - x_0 + x_0 e^{\mu_m t}}{x_m} \quad (4)$$

Nomenclature:

X is the biomass concentration (log10 CFU/ml); x_0 is the initial cell concentration; x_m is the maximum cell concentration; μ_m is the maximum specific growth rate (h^{-1}); *P* is the activity of enzyme (U, U/ml or U/L). P_0 is the initial activity of enzyme; α is the constant for enzyme accumulation decided by the cell growth rate; β is the constant for enzyme accumulation decided by the cell concentration. R^2 is correlation coefficients.

Results:

Fig. S1 The expression pattern of BgaB in mutant strains with different promoters. **(a)** strain BS43, **(b)** strain BSsrfA, **(c)** strain BSxylA without addition of xylose, **(d)** strain BSxylA with addition of 1% xylose after cultivation of 2 h**.** Biomass was plotted in logarithmic scale (logarithmic scale of biomass).

Fig. S2 The expression pattern of BgaB in mutant strains with different promoters. **(a)** strain BS43, **(b)** strain BSsrfA, **(c)** strain BSxylA without addition of xylose, **(d)** strain BSxylA with addition of 1% xylose after cultivation of 2 h**.** Biomass was plotted in logarithmic scale (linear scale of biomass).

Fig. S3 The expression pattern of BgaB controlled by mutant P_{ylb}. **(a)** strain WB800, **(b)** strain 35BgaB, **(c)** strain 10BgaB, **(d)** strain 3510BgaB, **(e)** strain 351022BgaB and **(f)** strain 351016BgaB (logarithmic scale of biomass).

Fig. S4 The expression pattern of BgaB controlled by mutant P_{ylb}. **(a)** strain WB800, **(b)** strain 35BgaB, **(c)** strain 10BgaB, **(d)** strain 3510BgaB, **(e)** strain 351022BgaB and **(f)** strain 351016BgaB (linear scale of biomass).

Fig. S5 The expression pattern of BgaB controlled by mutant *P3510*. **(a)** strain OBgaB, **(b)** strain JBgaB, **(c)** strain DBgaB, **(d)** strain BBgaB, **(e)** strain WBSBgaB and **(f)** strain NPBgaB (logarithmic scale of biomass).

Fig. S6 The expression pattern of BgaB controlled by mutant *P3510*. **(a)** strain OBgaB, **(b)** strain JBgaB, **(c)** strain DBgaB, **(d)** strain BBgaB, **(e)** strain WBSBgaB and **(f)** strain NPBgaB (linear scale of biomass).

Fig. S7 The expression pattern of sfGFP in mutant strains using P_{ylb} **(a)** and *NBP3510* **(b)**. **(a)** strain WBGFP, **(b)** strain WBSGFP (logarithmic scale of biomass).

Fig. S8 The expression pattern of sfGFP in mutant strains using P_{ylb} **(a)** and *NBP3510* **(b)**. **(a)** strain WBGFP, **(b)** strain WBSGFP (linear scale of biomass).

Fig. S9 The control of MPH **(a)** and Chd **(b)** expression in strain WB800. Biomass was plotted in logarithmic scale.

Fig. S10 The control of MPH **(a)** and Chd **(b)** expression in strain WB800. Biomass was plotted in linear scale.

Fig. S11 Purification of BgaB and sfGFP

Fig. S12 Purification of MPH and Chd

Fig. S13 Verification of BgaB, sfGFP, MPH and Chd by western blot

Fig. S14 The kinetics of bacterial growth. **(a)** strain WB800, **(b)** strain WBBgaB, **(c)** strain 35BgaB, **(d)** strain 10BgaB, **(e)** strain 3510BgaB, **(f)** strain 351022BgaB and **(g)** strain 351016BgaB.

Fig. S15 The kinetics of bacterial growth. **(a)** strain OBgaB, **(b)** strain JBgaB, **(c)** strain DBgaB, **(d)** strain BBgaB, **(e)** strain WBSBgaB and **(f)** strain NP3510BgaB.

Fig. S16 The kinetics of bacterial growth. **(a)** strain WB800, **(b)** strain WBSMPH and **(c)** strain WBSChd.

Fig. S17 The kinetic model for BgaB production in mutant strains. **(a)** strain WBBgaB, **(b)** strain WBSBgaB.

Table S1 Primers used in this study

Table S2 the parameters in the kinetics of bacterial growth

Table S3 the parameters in the kinetics of BgaB production

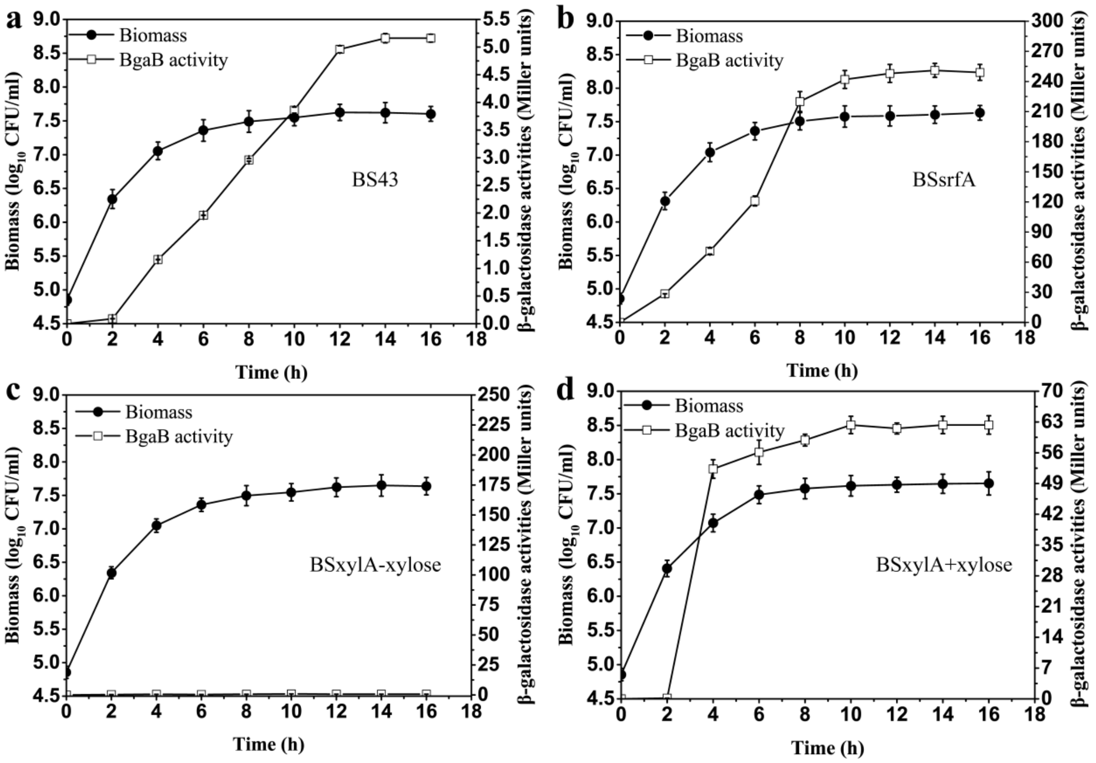

Fig. S1 The expression pattern of BgaB in mutant strains with different promoters. **(a)** strain BS43, **(b)** strain BSsrfA, **(c)** strain BSxylA without addition of xylose, **(d)** strain BSxylA with addition of 1% xylose after cultivation of 2 h**.** Biomass was plotted in logarithmic scale.

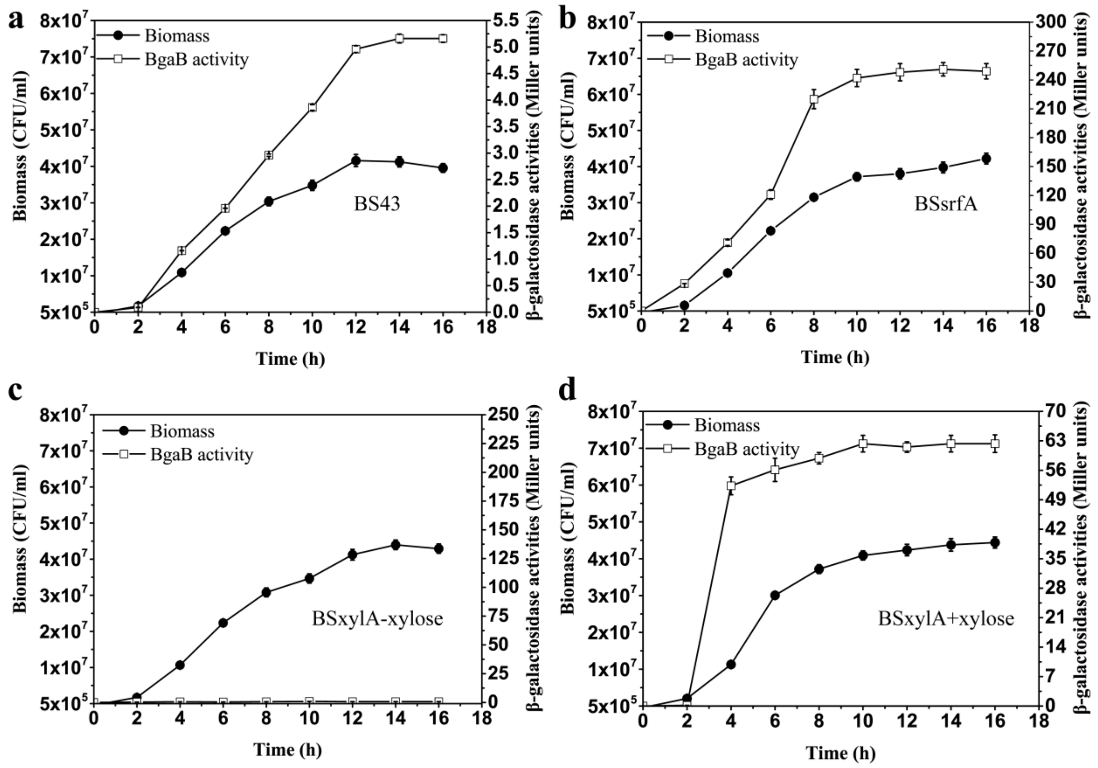

Fig. S2 The expression pattern of BgaB in mutant strains with different promoters. **(a)** strain BS43, **(b)** strain BSsrfA, **(c)** strain BSxylA without addition of xylose, **(d)** strain BSxylA with addition of 1% xylose after cultivation of 2 h**.** Biomass was plotted in linear scale.

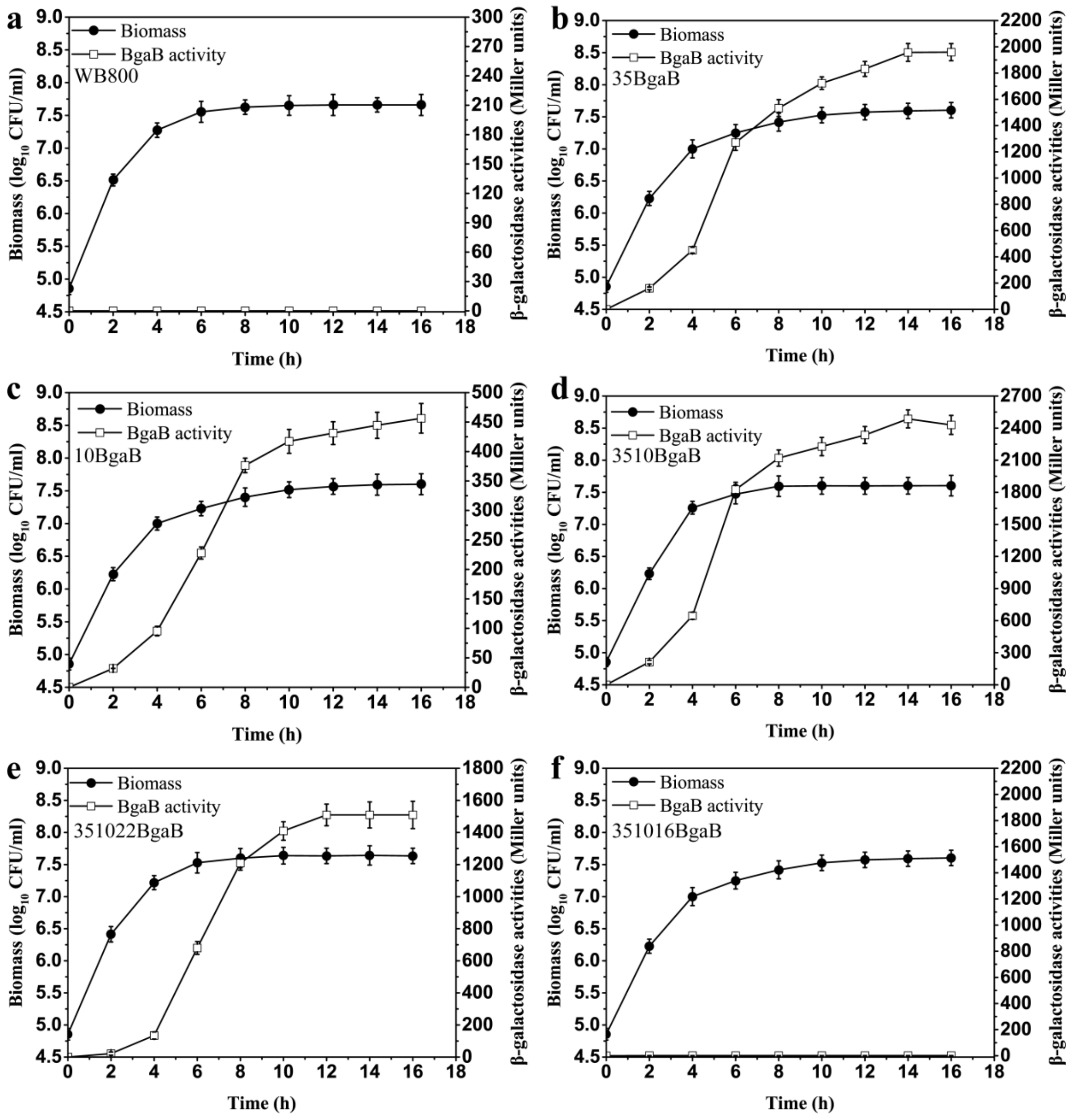

Fig. S3 The expression pattern of BgaB controlled by mutant P_{ylb}. **(a)** strain WB800, **(b)** strain 35BgaB, **(c)** strain 10BgaB, **(d)** strain 3510BgaB, **(e)** strain 351022BgaB and **(f)** strain 351016BgaB. Biomass was plotted in logarithmic scale.

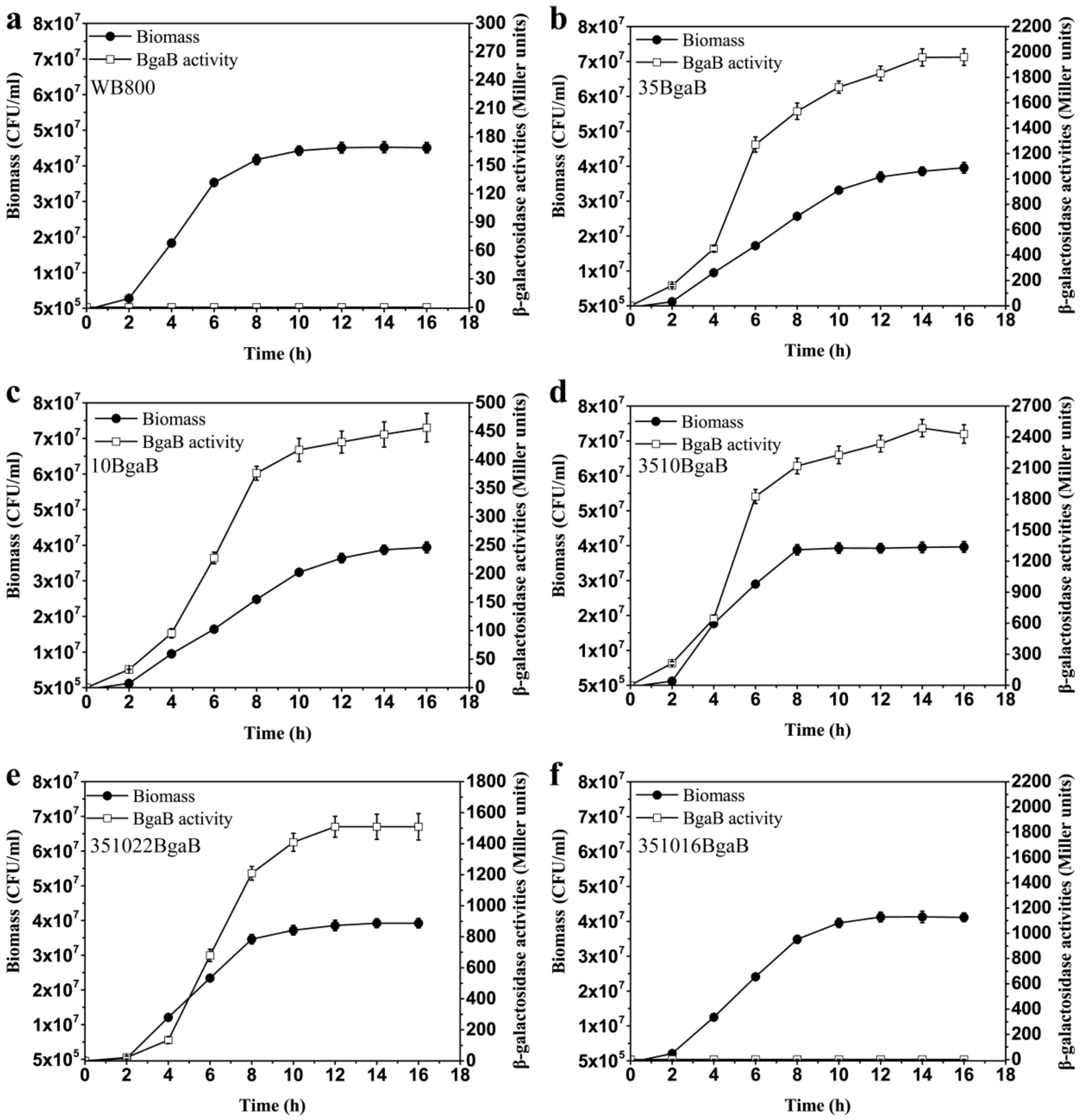

Fig. S4 The expression pattern of BgaB controlled by mutant P_{ylb}. **(a)** strain WB800, **(b)** strain 35BgaB, **(c)** strain 10BgaB, **(d)** strain 3510BgaB, **(e)** strain 351022BgaB and **(f)** strain 351016BgaB. Biomass was plotted in in linear scale.

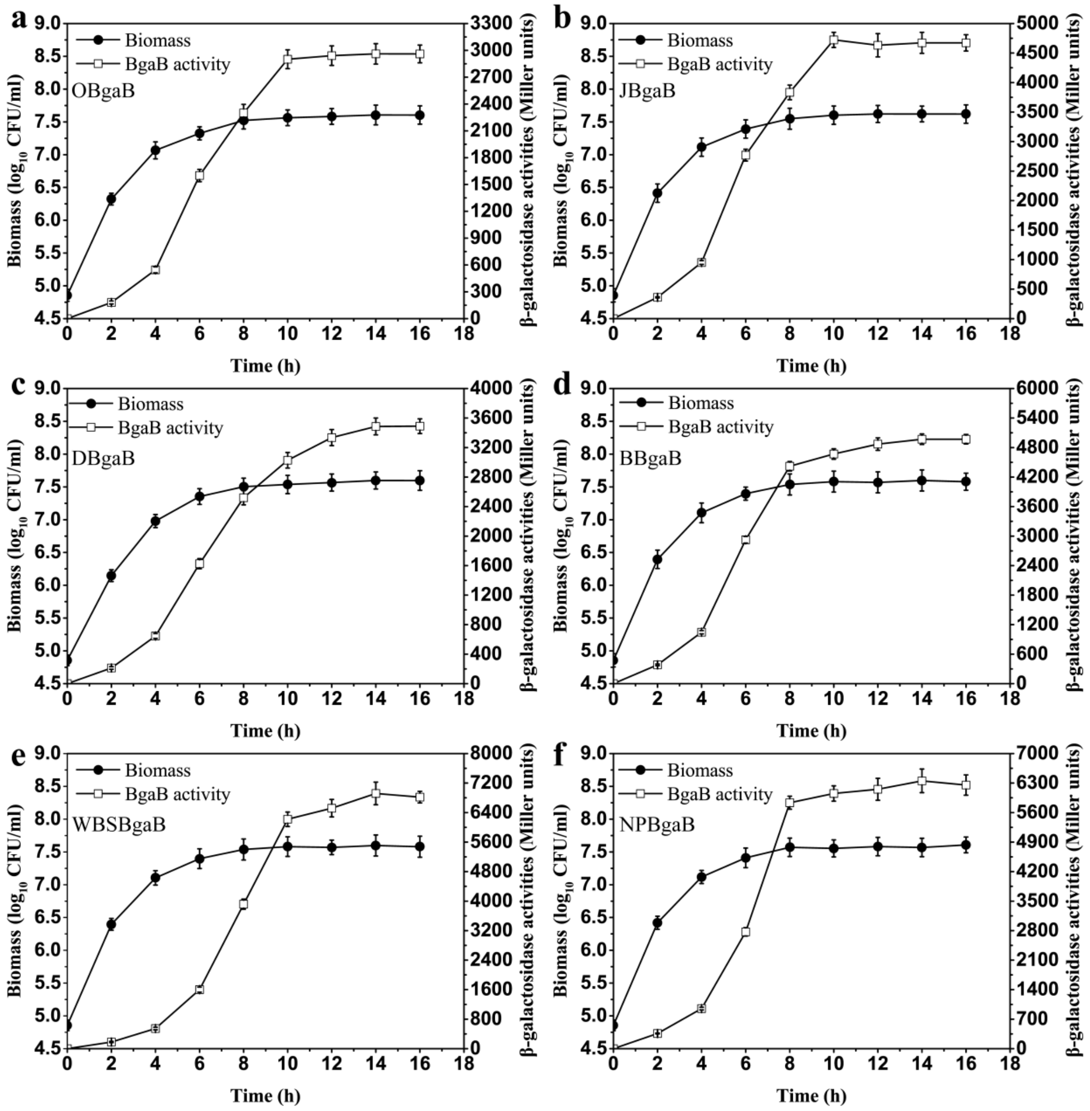

Fig. S5 The expression pattern of BgaB controlled by mutant *P3510*. **(a)** strain OBgaB, **(b)** strain JBgaB, **(c)** strain DBgaB, **(d)** strain BBgaB, **(e)** strain WBSBgaB and **(f)** strain NPBgaB. Biomass was plotted in logarithmic scale.

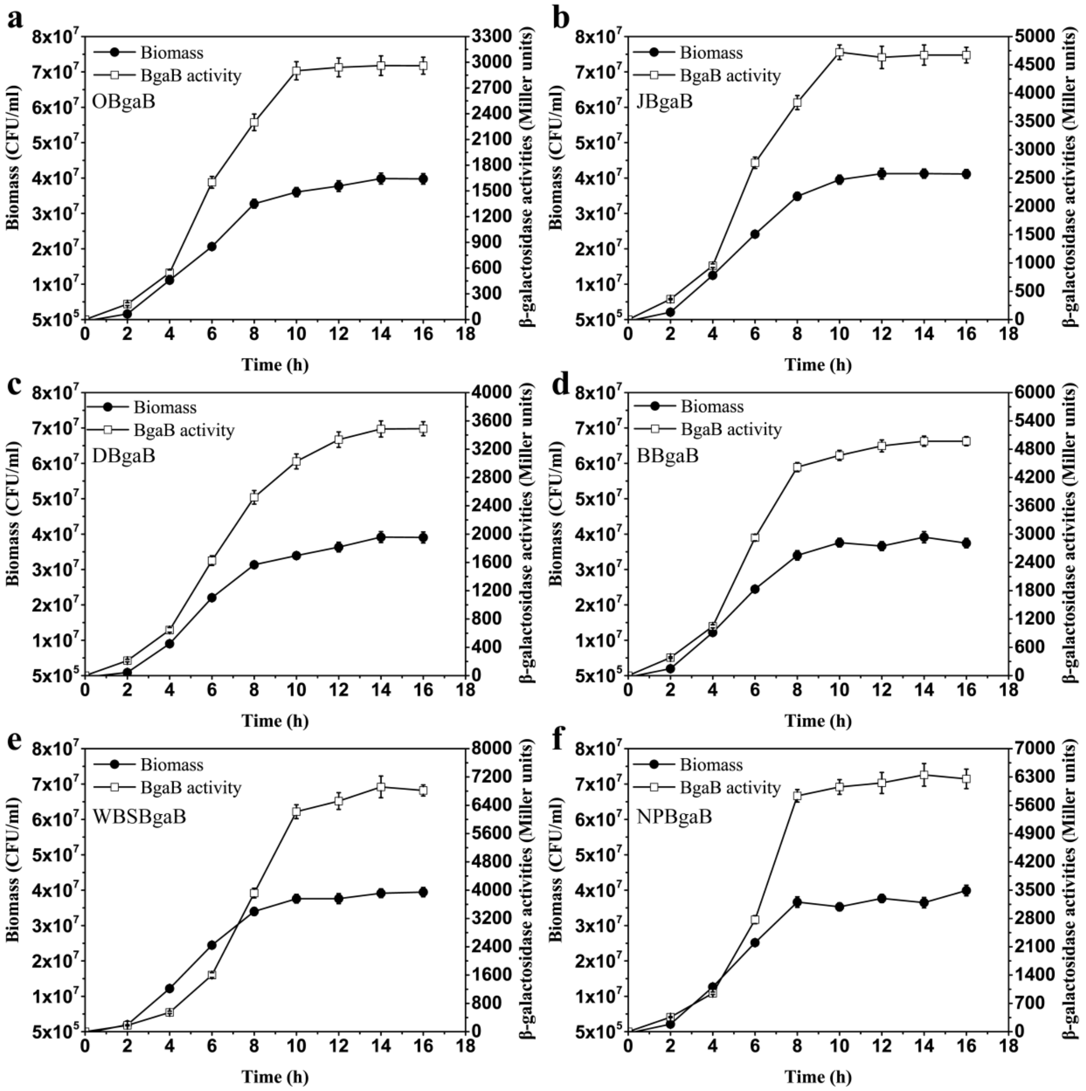

Fig. S6 The expression pattern of BgaB controlled by mutant *P3510*. **(a)** strain OBgaB, **(b)** strain JBgaB, **(c)** strain DBgaB, **(d)** strain BBgaB, **(e)** strain WBSBgaB and **(f)** strain NPBgaB. Biomass was plotted in linear scale.

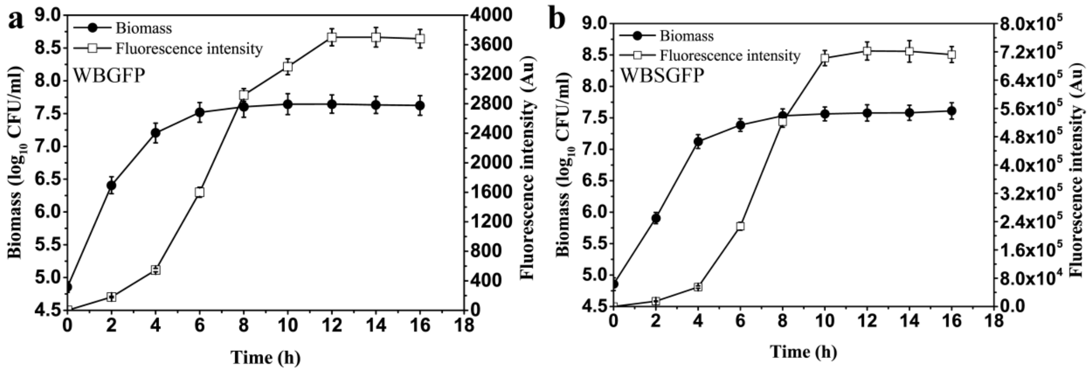

Fig. S7 The expression pattern of sfGFP in mutant strains using P_{ylb} **(a)** and *NBP3510* **(b)**. **(a)** strain WBGFP, **(b)** strain WBSGFP. Biomass was plotted in logarithmic scale.

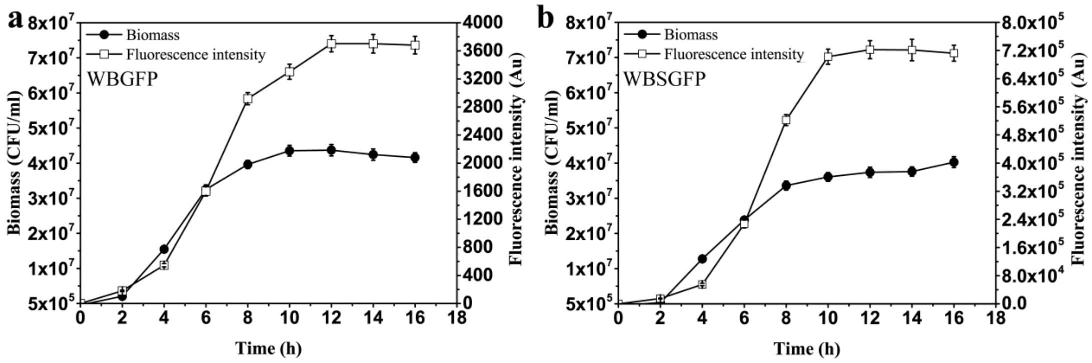

Fig. S8 The expression pattern of sfGFP in mutant strains using P_{ylb} **(a)** and *NBP3510* **(b)**. **(a)** strain WBGFP, **(b)** strain WBSGFP. Biomass was plotted in linear scale.

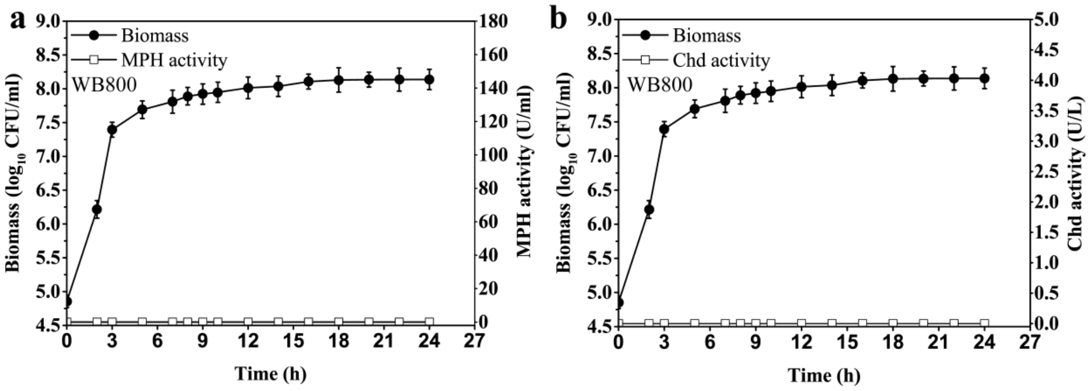

Fig. S9 The control of MPH **(a)** and Chd **(b)** expression in strain WB800. Biomass was plotted in logarithmic scale.

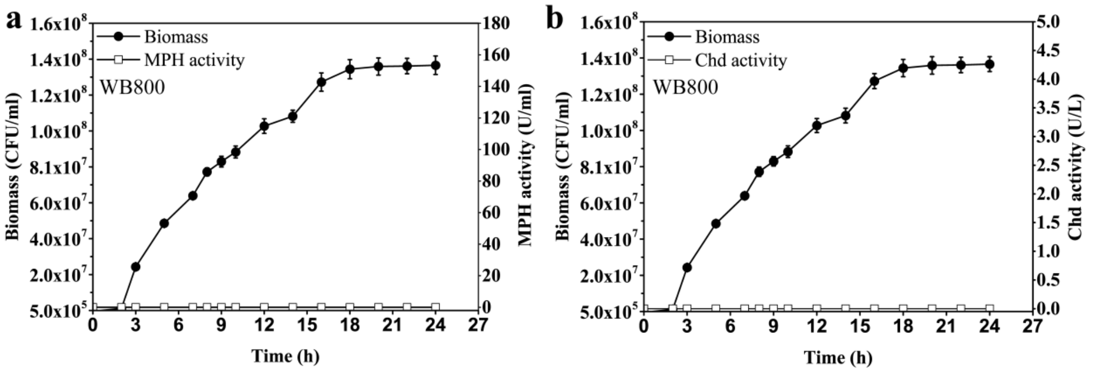

Fig. S10 The control of MPH **(a)** and Chd **(b)** expression in strain WB800. Biomass was plotted in linear scale.

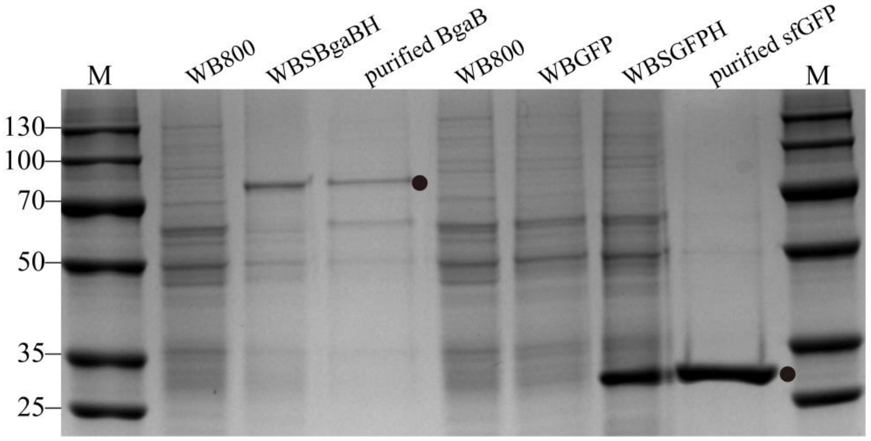

Fig. S11 Purification of BgaB and sfGFP. The bands indicating to BgaB and sfGFP were marked. WB800 represents the intracellular proteins of strain WB800.

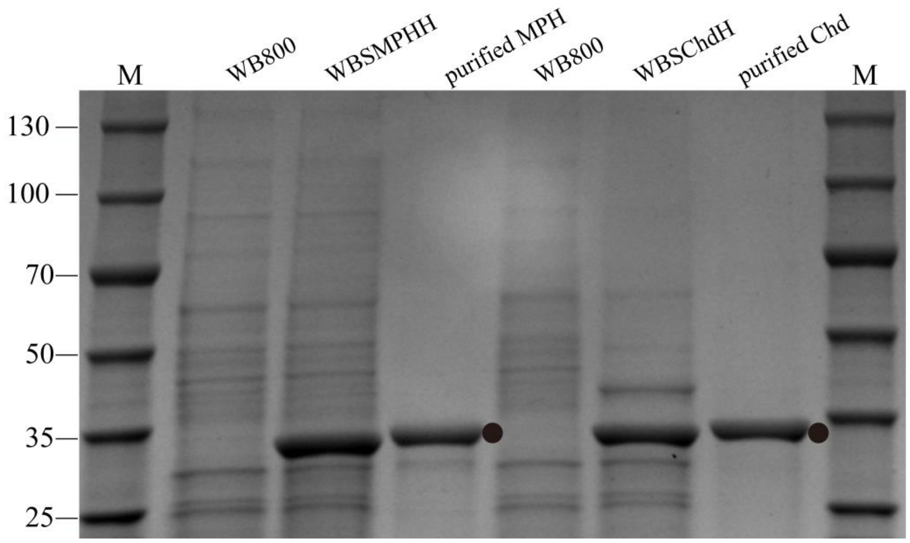

Fig. S12 Purification of MPH and Chd. The bands indicating to MPH and Chd were marked. WB800 represents the extracellular proteins of strain WB800.

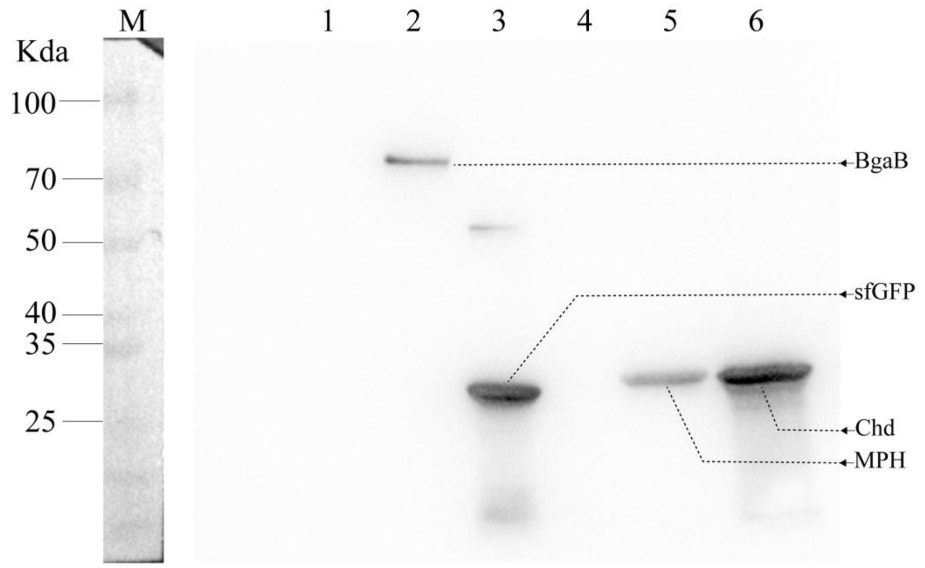

Fig. S13 Verification of BgaB (Lane 2), sfGFP (Lane 3), MPH (Lane 5) and Chd (Lane 6) by western blot. M, Marker. Lane 1, intracellular proteins of strain WB800. Lane 2, purified BgaB. Lane 3, purified sfGFP. Lane 4, extracellular proteins of strain WB800. Lane 5, purified MPH. Lane 6, purified Chd.

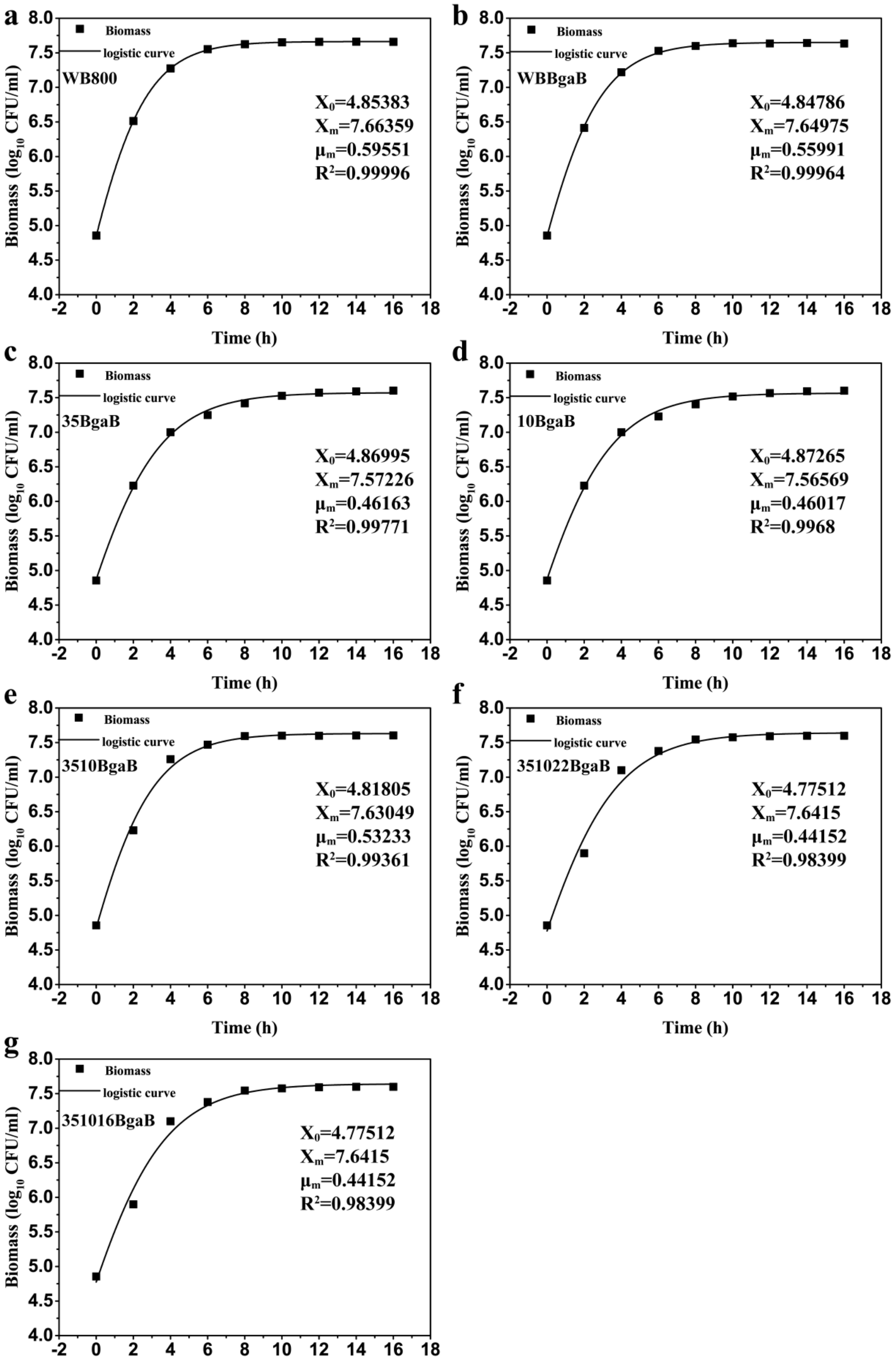

Fig. S14 The kinetics of bacterial growth. **(a)** strain WB800, **(b)** strain WBBgaB, **(c)** strain 35BgaB, **(d)** strain 10BgaB, **(e)** strain 3510BgaB, **(f)** strain 351022BgaB and **(g)** strain 351016BgaB.

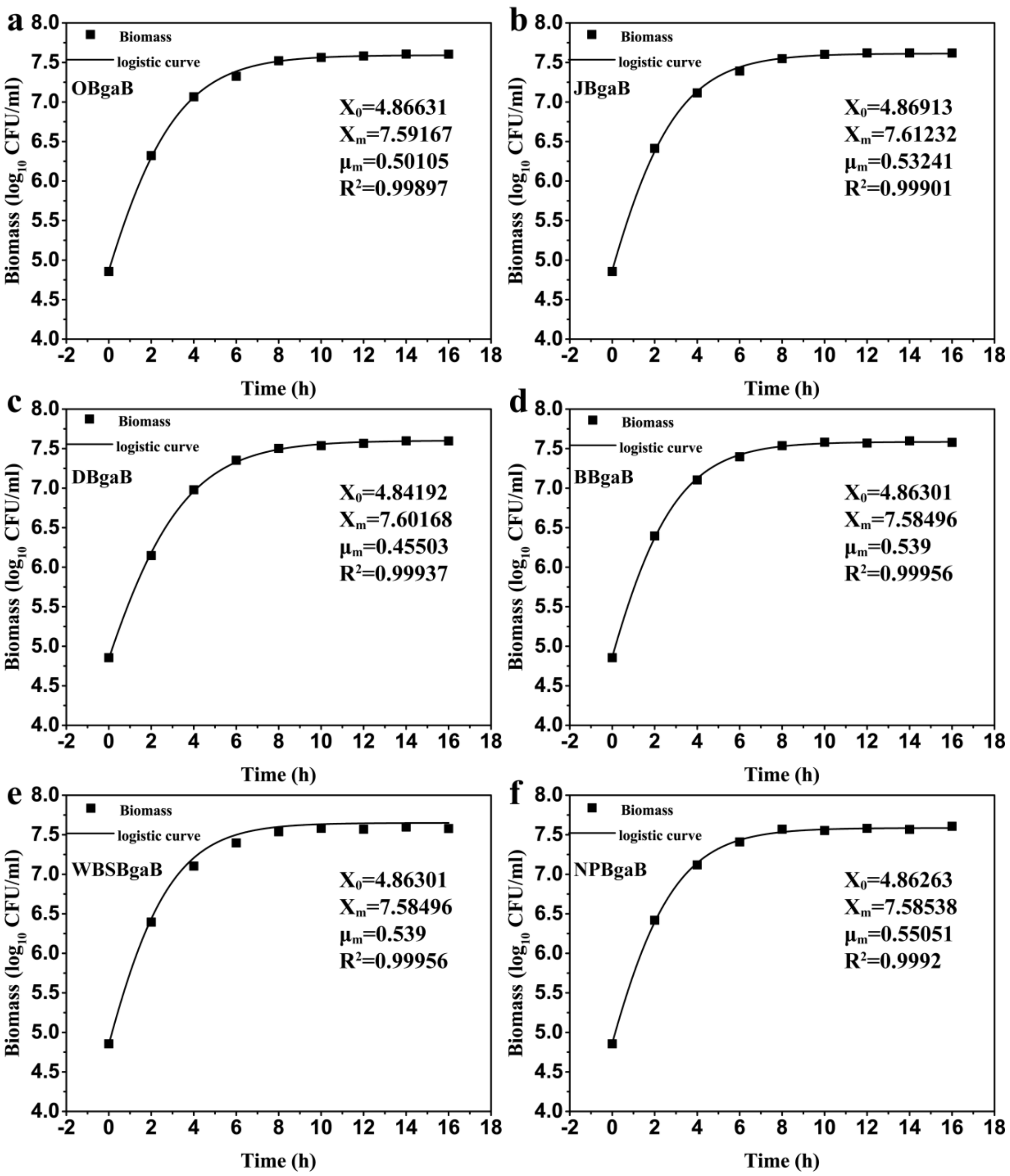

Fig. S15 The kinetics of bacterial growth. **(a)** strain OBgaB, **(b)** strain JBgaB, **(c)** strain DBgaB, **(d)** strain BBgaB, **(e)** strain WBSBgaB and **(f)** strain NP3510BgaB.

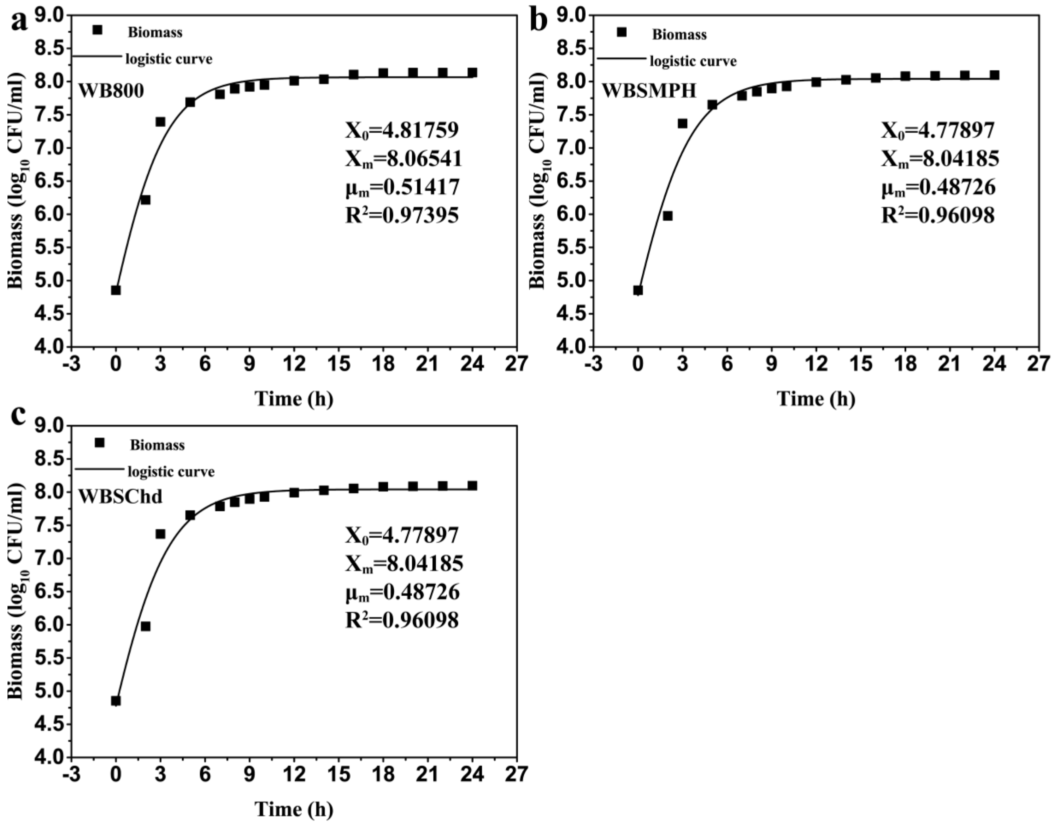

Fig. S16 The kinetics of bacterial growth. **(a)** strain WB800, **(b)** strain WBSMPH and **(c)** strain WBSChd.

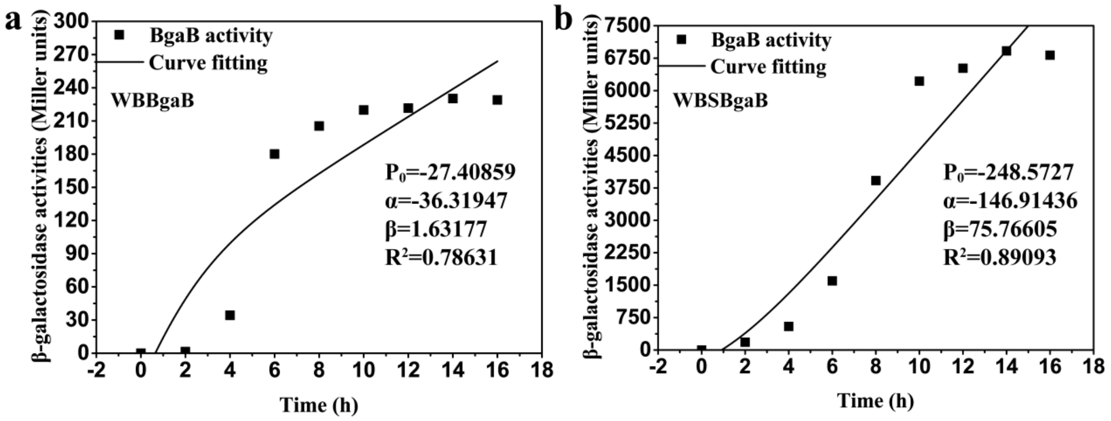

Fig. S17 The kinetic model for BgaB production in mutant strains. **(a)** strain WBBgaB, **(b)** strain WBSBgaB.

Table S1 Primers used in this study

Primers	Sequence (5’ to 3’)	Purpose
P1	tgtttgacaaaggtagaacgtatgaatgtgttatcctcaatttg	Amplification of promoter P_{ylb}
P2	tcctaacttataggggtaacacctaaaccttcccggcttcatca	Amplification of promoter P_{ylb}
P3	ttgtgctagaggatcaacttctcaaagatcccatgtgc	Amplification of *bgaB* gene
P4	caaattgaggataacacattcatacgttctacctttgtcaaaca	Amplification of *bgaB* gene
P5	gtgttacccctataagttagga	Amplification of pAX01 backbone
P6	tgatcctctagcacaaaagaa	Amplification of pAX01 backbone
P7	gcatccgacattcgatcattaca	upstream primer
P8	acatgggatctttgagaagtttctatgagtcgcttttgta	marker-free strain WBBgaB construction
P9	tacaaaagcgactcatagaaacttctcaaagatcccatgt	marker-free strain WBBgaB construction
P10	ttcgatggctttttgaacatca	downstream primer
P11	tacaaaagcgactcatagaatgataggtggtatgttttcg	Amplification of promoter P_{43}
P12	attgaggataacacattcatgcgtacattcctctcttaccca	Amplification of promoter P_{43}
P13	tacaaaagcgactcatagaattacattgtaatcatgtc	Amplification of promoter P_{xylA}
P14	attgaggataacacattcatgtgatttccccttaaaaataaattc	Amplification of promoter P_{xylA}
P15	tacaaaagcgactcatagaagacgctcttcgcaagggtgt	Amplification of promoter P_{srfA}
P16	attgaggataacacattcatattgtcatacctcccctaatc	Amplification of promoter P_{srfA}
P17	cgaaaacataccacctatcattctatgagtcgcttttgta	marker-free strain BS43 construction
P18	tgggtaagagaggaatgtacgcatgaatgtgttatcctcaat	marker-free strain BS43 construction
P19	gacatgattacaatgtaattctatgagtcgcttttgta	marker-free strain BSxylA construction
P20	gaatttatttttaaggggaaatcacatgaatgtgttatcctcaat	marker-free strain BSxylA construction
P21	acacccttgcgaagagcgtcttctatgagtcgcttttgta	marker-free strain WBsrfA construction
P22	gattaggggaggtatgacaatatgaatgtgttatcctcaat	marker-free strain WBsrfA construction
P23	taaacgctttatttaaaaatgtcaaatatttaaactttaattttaagc	marker-free strain 35BgaB construction
P24	gcttaaaattaaagtttaaatatttgacatttttaaataaagcgttta	marker-free strain 35BgaB construction
P25	ctttgttgtttctacatatattataaacgctttatttaaaaatgt	marker-free strain 10BgaB construction

P26	acatttttaaataaagcgtttataatatatgtagaaacaacaaag	marker-free strain 10BgaB construction
P27	ttgttgtttctacatatattataaacgctttatttaaaaatgtcaaatatttaaactt	marker-free strain 3510BgaB construction
P28	aagtttaaatatttgacatttttaaataaagcgtttataatatatgtagaaacaacaa	marker-free strain 3510BgaB construction
P29	ctccccctttgttgtttctacatatattataacatctttatttaaaaatgtcaaat	marker-free strain 351016BgaB construction
P30	atttgacatttttaaataaagatgttataatatatgtagaaacaacaaagggggag	marker-free strain 351016BgaB construction
P31	acatatattataaacgctttccctaaaaatgtcaaatatttaaac	marker-free strain 351022BgaB construction
P32	gtttaaatatttgacatttttagggaaagcgtttataatatatgt	marker-free strain 351022BgaB construction
P33	caaatattatgacacaaaaaagcgcagcatgggatctttgagaagtgttatcg	marker-free strain OBgaB construction
P34	tcccatgctgcgctttttgtgtcataatatttggatttttaaataaagcgtt	marker-free strain OBgaB construction
P35	caaatattttttgaagaaatactaaggatgggatctttgagaagtgttatcg	marker-free strain JBgaB construction
P36	tcccatccttagtatttcttcaaaaaaatatttggatttttaaataaagcgtt	marker-free strain JBgaB construction
P37	caaatactttttaaaagaatatcctcgatgggatctttgagaagtgttatcg	marker-free strain DBgaB construction
P38	tcccatcgaggatattcttttaaaaaagtatttggatttttaaataaagcgtt	marker-free strain DBgaB construction
P39	caaatatttttaaaaaaaatctataaaatgggatctttgagaagtgttatcg	marker-free strain BBgaB construction
P40	tcccattttatagatttttttaaaaaatatttggatttttaaataaagcgtt	marker-free strain BBgaB construction
P41	atttaaaaatgtcaaatatttttttaaaaaaaatttttaaatgggatctttgagaagt	marker-free strain WBSBgaB construction
P42	acttctcaaagatcccatttaaaaatttttttaaaaaaatatttgacatttttaaat	marker-free strain WBSBgaB construction
P43	gcaacatgtctgcgcaggctgcagcaccgcaggtgcgcac	Amplification of *mpd* gene
P44	tgactattctcagataaagttcatcatcacttggggttga	Amplification of *mpd* gene
P45	acatgtctgcgcaggctaccgaactcatcttggacttc	Amplification of *chd* gene
P46	ctagtaacatctgaccgagtcaaggcctggctgcgaga	Amplification of *chd* gene
P47	gtttgacaaaggtagaacgtatgagaagcaaaaaaattgtg	*aprE* signal peptide fused to *mpd*
P48	gtgcgcacctgcggtgctgcagcctgcgcagacatgttgc	*aprE* signal peptide fused to *mpd*
P49	gtttgacaaaggtagaacgtatgagaagcaaaaaaattgtg	*aprE* signal peptide fused to *chd*
P50	gaagtccaagatgagttcggtagcctgcgcagacatgt	*aprE* signal peptide fused to *chd*
P51	tgtttgacaaaggtagaacgtatgtcaaaaggagaagaact	marker-free strain WBGFP construction
P52	tatggacgaactttataaataagtgttacccctataagttaggagc	marker-free strain WBGFP construction

P53	tgtttgacaaaggtagaacgtatgtcaaaaggagaagaact	marker-free strain WBSGFP construction
P54	ggtatggacgaactttataaataagtgttacccctataagttaggagc	marker-free strain WBSGFP construction
P55	cacaatttttttgcttctcatacgttctacctttgtcaaac	marker-free strain WBSMPH construction
P56	tcaaccccaagtgatgatgaactttatctgagaatagtca	marker-free strain WBSMPH construction
P57	caattttttgcttctcatacgttctacctttgtcaaa	marker-free strain WBSChd construction
P58	tctcgcagccaggccttgaactttatctgagaatagtca	marker-free strain WBSChd construction
P59	agggaagttacacagttaggg	Construction of strain WBSBgaBH
P60	cgataacctagtggtggtggtggtggtgaaccttcccggcttcatc	Construction of strain WBSBgaBH
P61	aaggttcaccaccaccaccaccactaggttatcgattttcgttcgtg	Construction of strain WBSBgaBH
P62	ctccaaatatagcttgaaccgttcgtataatgtatgct	Construction of strain WBSBgaBH
P63	atacattatacgaacggttcaagctatatttggagttg	Construction of strain WBSBgaBH
P64	caggcatcgtggtgtca	Construction of strain WBSBgaBH
P65	ccttttctcttacttgttgcta	Construction of strain WBSGFPH
P66	taacacttagtggtggtggtggtggtgtttataaagttcgtccatacc	Construction of strain WBSGFPH
P67	ctttataaacaccaccaccaccaccactaagtgttacccctataagttaggagc	Construction of strain WBSGFPH
P68	gctgagattcgcccagg	Construction of strain WBSGFPH
P69	gattgggatgatagcgg	Construction of strain WBSMPHH
P70	catgggatctttgagaagttgatcctaagaatagtaatacaggat	Construction of strain WBSMPHH
P71	gtattactattcttaggatcaacttctcaaagatcccatgtg	Construction of strain WBSMPHH
P72	ttcatcatcagtggtggtggtggtggtgcttggggttgacgaccg	Construction of strain WBSMPHH
P73	caaccccaagcaccaccaccaccaccactgatgatgaactttatctgagaatag	Construction of strain WBSMPHH
P74	ccttccagggtatgtttct	Construction of strain WBSMPHH
P75	gattgggatgatagcgggagcat	Construction of strain WBSChdH
P76	ataaagttcagtggtggtggtggtggtgaggcctggctgcgagatc	Construction of strain WBSChdH
P77	caggcctcaccaccaccaccaccactgaactttatctgagaatagtcaatcttc	Construction of strain WBSChdH
P78	ccttccagggtatgtttctctttgat	Construction of strain WBSChdH

Table S2 the parameters in the kinetics of bacterial growth

Strains	Parameters[α]			
	X_0	X_m	μ_m	R^2
**Intracellular expression**				
WB800	4.85383	7.66359	0.59551	0.99996
WBBgaB	4.84786	7.64975	0.55991	0.99964
35BgaB	4.86995	7.57226	0.46163	0.99771
10BgaB	4.87265	7.56569	0.46017	0.9968
3510BgaB	4.81805	7.63049	0.53233	0.99361
351022BgaB	4.77512	7.6415	0.44152	0.98399
351016BgaB	4.77512	7.6415	0.44152	0.98399
OBgaB	4.86631	7.59167	0.50105	0.99897
JBgaB	4.86913	7.61232	0.53241	0.99901
DBgaB	4.84192	7.60168	0.45503	0.99937
BBgaB	4.86301	7.58496	0.53900	0.99956
NPBgaB	4.86263	7.58538	0.55051	0.9992
WBSBgaB	4.86301	7.58496	0.53900	0.99956
**Extracellular expression**				
WB800	4.81759	8.06541	0.51417	0.97395
WBSMPH	4.77897	8.04185	0.48726	0.96098
WBSChd	4.77897	8.04185	0.48726	0.96098

[α] X is the biomass concentration (log_{10} CFU/ml); X_0 is the initial cell concentration; X_m is the maximum cell concentration; μ_m is the maximum specific growth rate (h^{-1}); R^2 is correlation coefficients.

Table S3 the parameters in the kinetics of BgaB production

Strains	Parameters[α]			
	P_0	α	β	R^2
WBBgaB	-27.4086	-36.31947	1.63177	0.78631
WBSBgaB	-248.573	-146.9144	75.76605	0.89093

[α] *P* is the activity of enzyme (U, U/ml or U/L). P_0 is the initial activity of enzyme; α is the constant for enzyme accumulation decided by the cell growth rate; β is the constant for enzyme accumulation decided by the cell concentration. R^2 is correlation coefficients.

References:

1. Phisalaphong M, Srirattana N, Tanthapanichakoon W. Mathematical modeling to investigate temperature effect on kinetic parameters of ethanol fermentation**.** Biochem Eng J. 2006; 28**:**36-43.
2. Luedeking R, Piret EL. A kinetic study of the lactic acid fermentation. Batch process at controlled pH**.** BIOTECHNOL BIOENG. 2000; 67**:**636-644.
